# Development and validation of a simple machine learning tool to predict mortality in leptospirosis

**DOI:** 10.1038/s41598-023-31707-4

**Published:** 2023-03-18

**Authors:** Gabriela Studart Galdino, Tainá Veras de Sandes-Freitas, Luis Gustavo Modelli de Andrade, Caio Manuel Caetano Adamian, Gdayllon Cavalcante Meneses, Geraldo Bezerra da Silva Junior, Elizabeth de Francesco Daher

**Affiliations:** 1 Medical Sciences Postgraduate Program, Federal University of Ceará, Rua Silva Jatahy 1000 ap 600, Fortaleza, Ceará 60165-070 Brazil; 2 Hospital Universitário Walter Cantídio, Federal University of Ceará, Fortaleza, Ceará Brazil; 3 Hospital Geral de Fortaleza, Fortaleza, Ceara Brazil; 4 Botucatu Medical School, Universidade Estadual Paulista, Botucatu, São Paulo Brazil; 5 School of Medicine, Medical Sciences and Public Health Postgraduate Programs, University of Fortaleza, Fortaleza, Ceará Brazil

**Keywords:** Infectious diseases, Risk factors, Prognosis

## Abstract

Predicting risk factors for death in leptospirosis is challenging, and identifying high-risk patients is crucial as it might expedite the start of life-saving supportive care. Admission data of 295 leptospirosis patients were enrolled, and a machine-learning approach was used to fit models in a derivation cohort. The comparison of accuracy metrics was performed with two previous models—SPIRO score and quick SOFA score. A Lasso regression analysis was the selected model, demonstrating the best accuracy to predict mortality in leptospirosis [area under the curve (AUC-ROC) = 0.776]. A score-based prediction was carried out with the coefficients of this model and named LeptoScore. Then, to simplify the predictive tool, a new score was built by attributing points to the predictors with importance values higher than 1. The simplified score, named QuickLepto, has five variables (age > 40 years; lethargy; pulmonary symptom; mean arterial pressure < 80 mmHg and hematocrit < 30%) and good predictive accuracy (AUC-ROC = 0.788). LeptoScore and QuickLepto had better accuracy to predict mortality in patients with leptospirosis when compared to SPIRO score (AUC-ROC = 0.500) and quick SOFA score (AUC-ROC = 0.782). The main result is a new scoring system, the QuickLepto, that is a simple and useful tool to predict death in leptospirosis patients at hospital admission.

## Introduction

Leptospirosis is a worldwide neglected zoonotic disease caused by pathogenic spirochetes from the genus *Leptospira*, mainly *L. interrogans*, with higher prevalence in tropical countries^1^. It is a waterborne disease transmitted through rat urine, and its outbreaks occur during rainy seasons^2^. The disease mainly affects low-income populations and individuals exposed to contaminated animals and environments, such as farmers, veterinarians, sewage workers, meat inspectors, rodent control workers, and military personnel^2^.

Leptospirosis is a major concern for public health due to its high morbidity and mortality rates. It is estimated that 1.03 million new cases occur worldwide annually, along with 58,900 or even more deaths, mostly among young adult males^3^. Many cases of leptospirosis are undiagnosed or misdiagnosed as other tropical febrile illnesses, concealing its actual burden^4^. Therefore, the World Health Organization (WHO) considers leptospirosis an important neglected tropical zoonosis due to its underestimated incidence and high mortality^5^. The median mortality in a recent review was 10.05% [(range 0–33.3%)]^6^.

Most patients manifest an asymptomatic or mild disease with fever, chills, headache, and myalgia. Ocular and cutaneous manifestations have also been described^7,8^.

However, 10% of affected individuals may develop a life-threatening condition, characterized by hepatic dysfunction with rubinic jaundice and acute kidney injury (AKI), known as Weil’s syndrome, in addition to pulmonary hemorrhage, gastrointestinal symptoms, coagulopathy, electrolyte disturbances and myocarditis, as well as liver failure and neurological symptoms^4,9^.

The diagnosis of leptospirosis is challenging and cumbersome due to unspecific initial presentation that mimics other bacterial and viral infections, hampering early recognition, and the lack of a standard testing technique to check infection at all stages. Additionally, some endemic areas are unprovided of adequate laboratory resources and infrastructure as well as well trained staff^10^.

In an attempt to improve leptospirosis prognosis, previous studies have investigated risk factors for poor outcomes^6,11–14^. However, to the best of our knowledge, only one predictor of outcomes is available, SPIRO. This tool, built with data from patients hospitalized for leptospirosis in Australia, was based on three variables obtained at any time during hospitalization: abnormal auscultatory findings on respiratory examination, hypotension and oliguria. As limitations, no external validation was performed, and assessed end-point was a composite outcome of pulmonary hemorrhage, or intensive care unit (ICU) admission, or requirement for renal replacement therapy (RRT), or intubation, or need for vasoactive drugs^15^. Although these variables are classically associated with death, the indication for dialysis and ICU depend on the center's routine and logistics, which may preclude extrapolation to other centers. Thus, assessing death as an outcome would be more accurate. In the absence of specific predictors for leptospirosis, the classic predictors of death in septic patients have been used, such as the quick SOFA^16^. However, septic patients due to leptospirosis often have peculiar organic involvements that can have a distinct impact on outcomes.

Hence, aiming to assess a hard and objectively measurable endpoint, and focus on the possibility of early intervention, we proposed a new score constructed using machine learning techniques to predict death based on admission variable.

## Materials and methods

### Study design, population and ethics

This was a retrospective multicenter cohort study carried out from January 2005 to December 2019, including all patients with leptospirosis consecutively admitted to three tertiary reference hospitals in Fortaleza, state of Ceara, Brazil.

Patients with confirmed diagnosis of leptospirosis were included. The criteria for leptospirosis diagnosis included the presence of a positive serology result with a microscopic agglutination test (MAT) titer higher than 1:800, or ELISA assay for the detection of immunoglobulin M (IgM) antibodies associated with an epidemiological and clinical history compatible with leptospirosis. Patients with insufficient data for the diagnosis and those with concomitant acute infectious diseases (e.g., hepatitis A, HIV, dengue, typhoid fever) were excluded.

The study protocol was conducted in agreement with the Declaration of Helsinki and with resolution 466/2012 of the National Health Council, which regulates ethics in human research in Brazil. The Local Institutional Review Boards (IRB) of the three participating hospitals (Hospital São José de Doenças Infecciosas, Hospital Universitário Walter Cantídio, and Hospital Geral Fortaleza) have approved this study (no. 65452016.2.3001.5044). Due to the observational and retrospective nature of the study, using de-identified data, the IRBs waived the obtention of informed consent.

### Assessed parameters

Data were collected from the medical records, and patients were followed from hospital admission until death or hospital discharge, whichever comes first. Demographic and hospitalization characteristics, such as age, gender, the time between symptoms onset and hospital admission, and length of hospital stay were recorded. The clinical investigation included a record of clinical signs and symptoms presented at hospital admission, vital signs at admission (systolic and diastolic blood pressure, heart rate, and respiratory rate), acute kidney injury (AKI) development, and need for dialysis during hospitalization. Laboratory data collected within 24 h of hospital admission included serum urea, creatinine, sodium, potassium, direct bilirubin, indirect bilirubin, aspartate aminotransferase (AST), alanine aminotransferase (ALT), lactate dehydrogenase (LDH), creatine phosphokinase (CK), hemoglobin, hematocrit, white blood cell (WBC) count, platelet count, and arterial blood gas analysis.

AKI was defined according to the Kidney Disease Improving Global Outcomes (KDIGO) criteria^17^. Tachypnea was defined as a respiratory rate higher than 22 breaths per minute. Oliguria was defined as urine output < 400 mL/day after 24 h of effective hydration. Hypotension was defined as mean arterial blood pressure (MAP) < 60 mmHg, and therapy with vasoactive drugs was initiated when MAP remained lower than 60 mmHg despite the administration of parenteral fluids. Symptoms of pulmonary involvement were defined by the occurrence of coughing, crackles, or hemoptysis. Symptoms of lethargy were defined by the presence of sensory alterations, including disorientation, lethargy, and agitation.

### Outcome

The main evaluated outcome was in-hospital death.

### Statistical analysis

#### Exploratory data analysis

All variables of interest were compared between patients who survived and those who died during the hospital stay.

#### Predictive model—pre-processing step

We removed the variables with more than 30% of missing values (14% of the predictors) and imputed the others (Supporting information—S2-Table 2). A k-nearest neighbors (KNN) algorithm was used for the imputation method to account for missing values. All predictor variables were used to compute Gower's distance and the five nearest neighbors in the KNN imputation model. Once the nearest neighbors are determined, the model is used to impute nominal variables, and the mean is used for numerical data.

The continuous variables were standardized by subtracting their values from the mean (center) and dividing them by the standard deviation (scale). Continuous variables were transformed using Box–Cox transformation. Variables with zero or near-zero variance were removed from the model. In the feature engineering process for Lasso regression, natural splines with four degrees of freedom for age were chosen to account for the non-linearity.

For the class imbalance adjustment, the Synthetic Minority Over-sampling Technique (SMOTE) was used to create synthetic classes in the training set. The SMOTE algorithm generated new examples of the minority class using the nearest neighbors of these cases. This approach was used to balance the target class. All the pre-processing steps were performed in the training set.

### Feature selection

We used the Boruta algorithm to select the most important predictors. The Boruta algorithm is a feature selection method that classifies which features are important and which are not. The Boruta algorithm uses feature importance scores, which are provided by random forest. The importance measure of an attribute is obtained as the loss of classification accuracy caused by the random permutation of attribute values between objects. It is computed separately for all trees in the forest that use a given attribute for classification. Then the average and standard deviation of the accuracy loss are computed^18^. The method performs a top-down search for relevant features by comparing the importance of original attributes and progressively eliminating irrelevant features^19^. Features considered not important by the Boruta algorithm were removed. (Supporting information—S2-Table 2). We apply the feature selection in the training set.

### Model training

We split the data into derivation (training) and validation (test) datasets. To create the datasets, a random split was used, stratified by the target into training (80%) and test set (20%). In the training set (derivation cohort), bootstrap resampling was used to select the hyperparameters of the models and to reduce the bias.

We fitted gradient boosting decision trees (xgBoost), and Lasso regression to develop the candidate equations. Finally, the best hyperparameters were selected using machine learning approaches by bootstrap resampling in a training set aimed to maximize the area under the receiver operating characteristic (ROC) curve.

### Assessment of accuracy

The accuracy of the derivation cohort model was tested on the data of the validation cohort. The area under the ROC curve (AUC-ROC) was used to discriminate the ability of the models in the training and test sets. The 95% confidence interval (95%CI) of the AUC-ROC was estimated by bootstrap resampling (2000 samples) to reduce overfit bias. Additionally, the balanced accuracy, sensitivity and specificity were evaluated. Additionally, we estimate the best cut-point for ROC curve using the method of maximize the metric function, and J-Index metric using 1000 bootstrap resamples.

### Score fit and model visualization

The model with higher AUC-ROC in the validation cohort associated with better balanced accuracy values was used to build the new score named LeptoScore. Subsequently, a quick score (QuickLepto) was developed using the importance values of the highest coefficients of Lasso regression. For the development of QuickLepto for numerical predictors we discretized the data using the *cutoff* derived from a Classification and Regression Trees for Machine Learning (CART) tree.

### Accuracy metrics for previously published models

The final models (LeptoScore and QuickLepto) were compared with SPIRO and quick SOFA. SPIRO predict severe disease in patients with leptospirosis (pulmonary hemorrhage, or intensive care unit (ICU) admission, or requirement for renal replacement therapy (RRT), or intubation, or need for vasoactive drugs and is based on the following variables: oliguria (urine output ≤ 500 mL/24 h), abnormal auscultatory findings on respiratory examination and hypotension (systolic blood pressure ≤ 100 mmHg)^15^. The quick SOFA is a three-point score broadly used to identify high-risk patients for in-hospital mortality with suspected infection outside the ICU. Altered mental status (coma Glasgow score < 15), respiratory rate ≥ 22 breaths per minute and systolic BP ≤ 100 mmHg are the predictors of this score^16^. Given the relevance of these scores, we applied them (SPIRO and quick SOFA) in our dataset to compare the predictive values with the new LeptoScore and QuickLepto models.

The software R, version 4.0.2 and the tidymodels packages, and the R package “glmnet” statistical software (R Foundation) were used to perform the Lasso regression.

## Results

### Leptospirosis patients’ characteristics at hospital admission

A total of 295 leptospirosis patients were included. Death was observed in 32 cases (11%). The population was primarily young adults, with a median age of 36 years (25–49) and 86% were males. The median time from hospital admission to symptoms was 7 (5–8) days. Fever and chills were the most frequent symptoms (93%), followed by myalgia (78%) (Supporting information—S1-Table 1). The univariate analysis is shown in Supporting information—S1-Table 1.

### Predictive model

Patients were randomly grouped into two cohorts: the derivation cohort or training set (n = 235, 80%) and the internal validation cohort (test set) (n = 60, 20%).

There was a total of 63 predictors, and six were removed due to higher missing values (higher than 30%). Because there was a high number of predictors, feature selection was used, resulting in 14 possible candidate predictors. After that, three collinear predictors (Supporting information—S3-Table 3) were also removed**.** After that, predictive models were fitted using the final predictors (n = 11 predictors).

**Figure 1:**
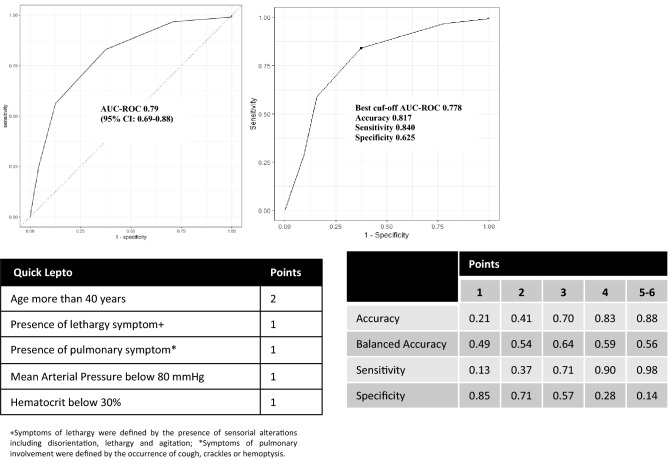
AUC-ROC curves of the QuickLepto in the validation cohort. A six-point scale and the discriminant measures of QuickLepto.

Several models were fitted with bootstrap resampling and the performance of these models was analyzed throughout the area under the curve of the receiver operating characteristic curves (AUC-ROC) in the derivation cohort. The AUC-ROC were 0.738, and 0.772 in the xgBoost and Lasso models, respectively. As a second step, the performance of these models was tested in the internal validation cohort. The AUC-ROC were 0.703 (0.414–0.987) and 0.776 (0.601–0.951) for the xgBoost and Lasso models, respectively (Table 1). The Lasso model had higher values of balance accuracy and specificity when compared to xgBoost (Table 1). Additionally, we plotted a confusion matrix of mortality in the derivative cohort as shown in Supporting information—S4-Fig. 1.

**Table 1:** Performance metrics of leptospirosis mortality models in the derivation and validation cohorts.

	Training set (n = 235)	Test set (n = 60)				
	AUC-ROC	AUC-ROC*	Accuracy	Balance Accuracy	Sensitivity	Specificity
Model						
xgBoost	0.738	0.703 (0.414–0.987)	0.883	0.686	0.943	0.428
Lasso	0.772	0.776 (0.601–0.951)	0.783	0.691	0.811	0.571

*[95% Confidence Interval] based on 2000 bootstrap resamples.

### Making a score-based prediction

The results of the Lasso model regression showed that older age, the lethargy symptom, pulmonary involvement symptom, higher alanine aminotransferase (ALT) values, higher direct bilirubin values, and higher leukocyte levels were related to death. In contrast, a higher hematocrit level, higher mean arterial pressure, higher urea and sodium values, and higher platelet levels were related to survival (Supporting information—S5-Fig. 2). The coefficients of the Lasso model were used to build the LeptoScore (Supporting information—S5-Fig. 2).

### Quick score (QuickLepto)—fit

To create QuickLepto, continuous variables were discretized based on a CART tree (Supporting information—S6-Fig. 3). Then, we used the coefficients of Lasso regression and mapped them in round numbers considering their absolute values. For variables whose Lasso regression coefficients were above 2, it was attributed 2 points (age); for those variables whose Lasso coefficients ranged from 0.5 and 2, it was given 1 point (pulmonary involvement, lethargy, hematocrit, and MAP). Those below 0.5 we excluded from QuickLepto (serum urea, sodium, bilirubin, ALT, leucocytes, platelets) (Supporting information—S7-Table 4).

The QuickLepto uses 5 predictors (Fig. 1):

1. Age over 40 years: 2 points
2. Presence of the lethargy symptom: 1 point
3. Presence of the pulmonary symptom: 1 point
4. Mean Arterial Pressure < 80 mmHg: 1 point
5. Hematocrit < 30%: 1 point

The AUC-ROC for QuickLepto was 0.788 [95% CI 0.693–0.883]. Accuracy, balanced accuracy, sensitivity, and specificity values using a cutoff of three or more points, are shown in Table 2, Fig. 1. The best cut-off for AUC-ROC was 0.778.

**Table 2:** Performance metrics of LeptoScore and QuickLepto in validation cohorts.

	Test set (n = 60)				
	AUC-ROC*	Accuracy	Balance Accuracy	Sensitivity	Specificity
Models					
LeptoScore	0.776 (0.601–0.951)	0.783	0.691	0.811	0.571
QuickLepto	0.788 (0.693–0.883)	0.700	0.644	0.716	0.571

*[95% Confidence Interval] based on 2000 bootstrap resamples.

### Comparison of accuracy metrics with previous models

The performances of LeptoScore and QuickLepto were compared with two other models, one derived from a population with leptospirosis and another model used in septic patients. The results are shown in Table 3. The accuracy, balanced accuracy, sensitivity, and specificity values were, respectively: 0.50, 0.71, 0.43, and 1.00 for the SPIRO score, and 0.78, 0.56, 0.84, and 0.28 for the model derived from quick SOFA. Therefore, all of them resulted in low specificity and/or lower sensitivity for patients with leptospirosis, showing a lower performance than the LeptoScore and QuickLepto score. Thus, the LeptoScore and QuickLepto score had a better balance between sensitivity and specificity.

**Table 3:** Comparison of accuracy metrics with the previous predictive model.

Model	Accuracy	Balance accuracy	Sensitivity	Specificity
SPIRO	0.500	0.761	0.433	1.00
Quick SOFA	0.782	0.567	0.849	0.285
LeptoScore (current model)	0.783	0.691	0.811	0.571
QuickLepto (current model)	0.700	0.644	0.716	0.571

## Discussion

This is the first study to predict mortality in human leptospirosis through a machine learning model in a high prevalence area, which was called LeptoScore. A quick score was also developed (QuickLepto), which could be easily applied and attained a similar performance to that of the complete model. Using age, two clinical symptoms, mean arterial pressure measure, and hematocrit values, it was possible to predict death at hospital admission with a high discriminatory power. Although many studies^11,12,14^, including a systematic review^6^, had found some independent predictors of mortality, this is the first study that established a hospital admission tool that is easy to use and has the best balance between sensitivity and specificity to predict death in human leptospirosis.

Previous predictive models in leptospirosis showed high performance but used combined data obtained at admission and other moments during hospital stay. For example, the SPIRO score predicts leptospirosis severity using: oliguria (urine output ≤ 500 mL/24 h), abnormal auscultatory findings on respiratory examination and hypotension (systolic blood pressure ≤ 100 mmHg)^15^. The presence of oliguria must be evaluated 24 h after and not promptly at hospital admission. Aiming to focus on early intervention, the new score was developed using admission variables and evaluated a hard and objectively measurable endpoint, death.

The present study had 11% of mortality (32/295), a similar finding to that of previous studies, which was around 10% (range of 0–33.3%)^6^. Independent risk factors for mortality in leptospirosis-associated AKI reported in a systematic review were oliguria, jaundice, arrhythmia, crackles, elevated direct bilirubin level, elevated activated prothrombin time, hyperbilirubinemia and leukocytosis^6,20^. We found similar mortality predictors in the present cohort, not exclusively related to the presence of AKI.

A study conducted in patients with leptospirosis in intensive care units showed that the Simplified Acute Physiology Score (SAPS) showed a worse performance in relation to mortality. The mortality of patients with leptospirosis was lower than that predicted by the SAPS score^21^. This suggests the need for a specific predictive model for patients with leptospirosis. Confirming these findings, the quick SOFA, which was a general score for septic patients, showed an inferior performance than the LeptoScore.

Three of the five predictors included in QuickLepto are similar to the parameters used in quick SOFA, but anemia (Hematocrit < 30%) and age over 40 years, the most important variables, were not included.

In line with previous studies, our results showed that anemia in leptospirosis patients was associated with poor outcomes. In a prospective observational study, hemoglobin (Hb) levels lower than 11 g/dL were associated with severe forms of the disease (70% versus 14.8%; OR = 16.2 [95% CI 3.9–66.9])^22^. Another study has shown that ICU patients with leptospirosis had lower levels of hemoglobin than those treated in hospital wards (10.2 ± 2.4 vs. 11.6 ± 1.9 g/dL, p < 0.0001)^23^.

Daher et al. have previously shown that age is a crucial predictor of outcomes. Elderly patients with leptospirosis showed less hemodynamic impairment on admission, higher incidence of AKI (OR 2.049, 95% CI 1.207–3.477), and a higher frequency of death (OR 3.520, 95% CI 1.940–6.386) during hospital stay than younger patients^13^.

This study has some limitations that are mainly due to its retrospective design and the fact that data were collected over 14 years. Although the long period, the main treatment guidelines remain the same. Our previous article showed that the mortality rate had dropped each decade since 1985, which probably reflects early diagnosis and the provision of adequate treatment. The QuickLepto did not have an external cohort validation. Although we performed the validation metrics in an independent test set, the results of QuickLepto need further external validation cohorts. This was especially true for the patients that scored more than 3 points because the number of patients that ranked 4–5 points was lower in the present dataset. On the other hand, only basic hospital admission data were included, the statistical models used in the study are very sophisticated, and it is one of the largest samples ever studied.

In conclusion, patient age, presence of lethargy or pulmonary symptoms, arterial hypotension, and anemia were associated with death in patients with leptospirosis requiring hospitalization. These variables were selected to fit a new scoring system, the QuickLepto, a simple and useful tool to predict death in leptospirosis patients at hospital admission. Despite its good accuracy in predicting death, the LeptoScore is more complex, requiring specific calculators. Thus, we encourage physicians in the clinical setting to use QuickLepto to predict outcomes and make decisions, such as choosing the appropriate ward, allocating staff, and prescribing treatments and interventions. The next step is its validation in a prospective sample, especially in different populations, to provide overall appropriateness and demonstrate its significant usefulness in resource-limited settings with the greatest clinical burden.

## Supplementary Information

Supplementary Information.

## Data Availability

The dataset supporting the conclusions of this article is available upon reasonable request from the corresponding author (GSG).
