# Supplementary material for: Development and validation of a simple machine learning tool to predict mortality in leptospirosis
Source: Sci Rep. 2023 Mar 18;13:4506. doi: 10.1038/s41598-023-31707-4 (PMC10024714; doi:10.1038/s41598-023-31707-4)
Supplement: Supplementary file 1 — Supplementary Information. [file 41598_2023_31707_MOESM1_ESM.docx]

**Supporting Information**

**Supplemental Tables and Figures**

**S 1 - Table 1.** Baseline characteristics of patients with leptospirosis

**S 2 - Table 2.** Missing Data

**S 3 - Table 3.** Feature Selection by Boruta

**S 4 - Figure 1.** Confusion Matrix of the results of the xgBoost (A) and the Lasso model (B) in the validation cohort

**S 5 - Figure 2.** Variable importance associated with death in leptospirosis patients (Lepto Score)

**S 6 - Figure 3.** Classification And Regression Trees for Machine Learning (CART) Plot

**S7 -** **Table 4.** Coefficients of Lasso regression (final model)

**S1 - Table 1. Baseline characteristics of patients with leptospirosis divided by the outcome of death**

| **Characteristic** | **Overall**  **(N = 295)** |
| --- | --- |
| Age (years) | 36 (25, 49) |
| **Gender** |  |
| Male (%) | 248 (84%) |
| Female (%) | 47 (16%) |
| **Occupation** |  |
| Farmer | 29 (12%) |
| Other | 204 (88%) |
| *Missing* | 62 |
| Time between symptom onset and hospitalization (days) | 7 (5, 8) |
| *Missing* | 45 |
| **Signs and Symptoms** |  |
| Lethargy | 30 (11%) |
| *Missing* | 12 |
| Fever or Chills | 263 (93%) |
| *Missing* | 12 |
| Vomiting | 160 (57%) |
| *Missing* | 12 |
| Cholestatic Syndrome (Jaundice or Choluria) | 185 (65%) |
| *Missing* | 12 |
| Myalgia | 220 (78%) |
| *Missing* | 12 |
| Rash | 45 (16%) |
| *Missing* | 12 |
| Minor Bleeding (Epistaxis or Gum Bleeding) | 13 (4.6%) |
| *Missing* | 12 |
| Dizziness | 67 (24%) |
| *Missing* | 12 |
| Hepatomegaly | 43 (15%) |
| *Missing* | 12 |
| Headache | 168 (59%) |
| *Missing* | 12 |
| Pulmonary involvement (Cough, Hemoptysis or Crackles) | 130 (46%) |
| *Missing* | 12 |
| Dehydration | 59 (21%) |
| *Missing* | 12 |
| Diarrhea | 106 (37%) |
| *Missing* | 12 |
| Abdominal Pain | 118 (42%) |
| *Missing* | 12 |
| Calf Pain | 137 (55%) |
| *Missing* | 48 |
| Cavity Spilling | 40 (14%) |
| *Missing* | 12 |
| Bruises | 4 (1.4%) |
| *Missing* | 12 |
| Hematemesis | 16 (5.7%) |
| *Missing* | 13 |
| Melena | 10 (3.5%) |
| *Missing* | 12 |
| Metrorrhagia | 3 (1.1%) |
| *Missing* | 12 |
| Hematuria | 22 (7.8%) |
| *Missing* | 12 |
| Hypotension | 32 (11%) |
| *Missing* | 12 |
| Secondary Infection | 15 (5.3%) |
| *Missing* | 12 |
| Insomnia | 7 (2.5%) |
| *Missing* | 12 |
| Reduced Diuresis | 79 (28%) |
| *Missing* | 13 |
| Pallor | 53 (19%) |
| *Missing* | 12 |
| Petechiae | 11 (3.9%) |
| *Missing* | 12 |
| Conjunctival Suffusion | 37 (13%) |
| *Missing* | 12 |
| **Vital Signs at Admission** |  |
| Systolic Pressure (mmHg) | 114 (100, 130) |
| *Missing* | 37 |
| Diastolic Pressure (mmHg) | 70 (60, 80) |
| *Missing* | 41 |
| MAP (mmHg) | 87 (75, 97) |
| *Missing* | 47 |
| Heart Rate (/min) | 96 (81, 108) |
| *Missing* | 52 |
| Respiratory Rate (/min) | 22 (20, 28) |
| *Missing* | 76 |
| **Laboratory Data** |  |
| Urea (mg/dl) | 83 (42, 132) |
| *Missing* | 36 |
| Creatinine (mg/dl) | 2.44 (1.20, 4.30) |
| *Missing* | 18 |
| Sodium (mEq/l) | 135.0 (131.0, 138.0) |
| *Missing* | 57 |
| Potassium (mEq/l) | 3.80 (3.30, 4.30) |
| *Missing* | 40 |
| Direct bilirubin (mg/dl) | 3.7 (0.7, 10.0) |
| *Missing* | 86 |
| Indirect bilirubin (mg/dl) | 1.1 (0.5, 3.2) |
| *Missing* | 87 |
| AST (UI/l) | 88 (47, 186) |
| *Missing* | 53 |
| ALT (UI/l) | 68 (38, 114) |
| *Missing* | 55 |
| Hematocrit (%) | 34 (30, 38) |
| *Missing* | 12 |
| Hemoglobin (g/dl) | 11.60 (10.07, 12.70) |
| *Missing* | 23 |
| WBC (10^9^/l) | 11,400 (7,990, 15,615) |
| *Missing* | 11 |
| Platelets (10^9^/l) | 78,000 (43,000, 157,000) |
| *Missing* | 11 |

MAP, mean arterial pressure; AST, aspartate aminotransferase; ALT, alanine aminotransferase; WBC, white blood cell count.

**S 2 - Table 2. Missing Data**

| Variable | Variable Type | N | N missing | Missing Percent |
| --- | --- | --- | --- | --- |
| age | Predictor | 295 | 0 | 0.0 |
| sex | Predictor | 295 | 0 | 0.0 |
| ocupation | Predictor | 233 | 62 | 21.0 |
| time_symptoms_admission | Predictor | 250 | 45 | 15.3 |
| symptom_letargy | Predictor | 283 | 12 | 4.1 |
| symptom_fever_chills | Predictor | 283 | 12 | 4.1 |
| symptom_vomit | Predictor | 283 | 12 | 4.1 |
| symptom_palpitation | Predictor | 196 | 99 | 33.6 |
| symptom_cholestatic_syndrome | Predictor | 283 | 12 | 4.1 |
| symptom_tachypneia | Predictor | 108 | 187 | 63.4 |
| symptom_myalgia | Predictor | 283 | 12 | 4.1 |
| symptom_rash | Predictor | 283 | 12 | 4.1 |
| symptom_minor_bleeding | Predictor | 283 | 12 | 4.1 |
| symptom_dizziness | Predictor | 283 | 12 | 4.1 |
| symptom_hepatomegaly | Predictor | 283 | 12 | 4.1 |
| symptom_headache | Predictor | 283 | 12 | 4.1 |
| symptom_convulsions | Predictor | 170 | 125 | 42.4 |
| symptom_pulmonary_involvement | Predictor | 283 | 12 | 4.1 |
| symptom_dehydratation | Predictor | 283 | 12 | 4.1 |
| symptom_diarrhea | Predictor | 283 | 12 | 4.1 |
| symptom_abdominal_pain | Predictor | 283 | 12 | 4.1 |
| symptom_calf_pain | Predictor | 247 | 48 | 16.3 |
| symptom_cavity_spill | Predictor | 283 | 12 | 4.1 |
| symptom_bruises | Predictor | 283 | 12 | 4.1 |
| symptom_Flapping | Predictor | 170 | 125 | 42.4 |
| symptom_hematemesis | Predictor | 282 | 13 | 4.4 |
| symptom_melena | Predictor | 283 | 12 | 4.1 |
| symptom_metrorrhagia | Predictor | 283 | 12 | 4.1 |
| symptom_hematuria | Predictor | 283 | 12 | 4.1 |
| symptom_hypotension | Predictor | 283 | 12 | 4.1 |
| symptom_secondary_infection | Predictor | 283 | 12 | 4.1 |
| symptom_insomnia | Predictor | 283 | 12 | 4.1 |
| symptom_reduced_diuresis | Predictor | 282 | 13 | 4.4 |
| symptom_pallor | Predictor | 283 | 12 | 4.1 |
| symptom_pancreatitis | Predictor | 175 | 120 | 40.7 |
| symptom_petequias | Predictor | 283 | 12 | 4.1 |
| symptom_polyuria | Predictor | 168 | 127 | 43.1 |
| symptom_conjuntival_suffusion | Predictor | 283 | 12 | 4.1 |
| vital_Pressure_systolic_admission | Predictor | 258 | 37 | 12.5 |
| vital_Pressure_diastolic_admission | Predictor | 254 | 41 | 13.9 |
| vital_HR_admission | Predictor | 243 | 52 | 17.6 |
| vital_RR_admission | Predictor | 219 | 76 | 25.8 |
| MAP | Predictor | 248 | 47 | 15.9 |
| vasopressor | Predictor | 236 | 59 | 20.0 |
| diuretic | Predictor | 234 | 61 | 20.7 |
| blood_transfussion | Predictor | 232 | 63 | 21.4 |
| antibiotics | Predictor | 295 | 0 | 0.0 |
| lab_urea | Predictor | 259 | 36 | 12.2 |
| lab_dela_urea | Predictor | 144 | 151 | 51.2 |
| lab_creatinine | Predictor | 277 | 18 | 6.1 |
| lab_sodium | Predictor | 238 | 57 | 19.3 |
| lab_potassium | Predictor | 255 | 40 | 13.6 |
| lab_bicarbonate | Predictor | 151 | 144 | 48.8 |
| lab_pH | Predictor | 155 | 140 | 47.5 |
| lab_bilirubin_D | Predictor | 209 | 86 | 29.2 |
| lab_bilirubin_I | Predictor | 208 | 87 | 29.5 |
| lab_TGO | Predictor | 242 | 53 | 18.0 |
| lab_TGP | Predictor | 240 | 55 | 18.6 |
| lab_HTc | Predictor | 283 | 12 | 4.1 |
| lab_Hb | Predictor | 272 | 23 | 7.8 |
| lab_leukocytes | Predictor | 284 | 11 | 3.7 |
| lab_platelets | Predictor | 284 | 11 | 3.7 |
| length_stay | Predictor | 262 | 33 | 11.2 |
| hemodialysis | Outcome | 295 | 0 | 0.0 |
| N_dialysis_section | Outcome | 128 | 167 | 56.6 |
| dialysis_type | Outcome | 100 | 195 | 66.1 |
| AKI | Outcome | 295 | 0 | 0.0 |
| ICU | Outcome | 28 | 267 | 90.5 |
| KDIGO | Outcome | 295 | 0 | 0.0 |
| KDIGO3 | Outcome | 295 | 0 | 0.0 |
| death | Outcome | 295 | 0 | 0.0 |
| composite_outcome | Outcome | 295 | 0 | 0.0 |
| spiro_score | Outcome | 295 | 0 | 0.0 |

Total predictors: 63; Total Outcome: 10

Remove more than 30% missing variables (9/63 predictors):

1. symptom_tachypneia

2. symptom_palpitation

3. symptom_convulsions

4. symptom_Flapping

5. symptom_pancreatitis

6. symptom_polyuria

7. lab_dela_urea

8. lab_bicarbonate

9. lab_pH

**S3 - Table 3. Feature Selection by Boruta**

| Variable | Variable Type | N | Classification | Retain Model |
| --- | --- | --- | --- | --- |
| age | Predictor | 295 | Confirmed | Yes |
| sex | Predictor | 295 | Rejected | No |
| ocupation | Predictor | 233 | Rejected | No |
| time_symptoms_admission | Predictor | 250 | Rejected | No |
| symptom_letargy | Predictor | 283 | Tentative | Yes |
| symptom_fever_chills | Predictor | 283 | Rejected | No |
| symptom_vomit | Predictor | 283 | Rejected | No |
| symptom_palpitation | Predictor | 196 | Rejected | No |
| symptom_cholestatic_syndrome | Predictor | 283 | Rejected | No |
| symptom_tachypneia | Predictor | 108 | Rejected | No |
| symptom_myalgia | Predictor | 283 | Rejected | No |
| symptom_rash | Predictor | 283 | Rejected | No |
| symptom_minor_bleeding | Predictor | 283 | Rejected | No |
| symptom_dizziness | Predictor | 283 | Rejected | No |
| symptom_hepatomegaly | Predictor | 283 | Rejected | No |
| symptom_headache | Predictor | 283 | Rejected | No |
| symptom_convulsions | Predictor | 170 | Rejected | No |
| symptom_pulmonary_involvement | Predictor | 283 | Confirmed | Yes |
| symptom_dehydratation | Predictor | 283 | Rejected | No |
| symptom_diarrhea | Predictor | 283 | Rejected | No |
| symptom_abdominal_pain | Predictor | 283 | Rejected | No |
| symptom_calf_pain | Predictor | 247 | Rejected | No |
| symptom_cavity_spill | Predictor | 283 | Rejected | No |
| symptom_bruises | Predictor | 283 | Rejected | No |
| symptom_Flapping | Predictor | 170 | Rejected | No |
| symptom_hematemesis | Predictor | 282 | Rejected | No |
| symptom_melena | Predictor | 283 | Rejected | No |
| symptom_metrorrhagia | Predictor | 283 | Rejected | No |
| symptom_hematuria | Predictor | 283 | Rejected | No |
| symptom_hypotension | Predictor | 283 | Rejected | No |
| symptom_secondary_infection | Predictor | 283 | Rejected | No |
| symptom_insomnia | Predictor | 283 | Rejected | No |
| symptom_reduced_diuresis | Predictor | 282 | Rejected | No |
| symptom_pallor | Predictor | 283 | Rejected | No |
| symptom_pancreatitis | Predictor | 175 | Rejected | No |
| symptom_petequias | Predictor | 283 | Rejected | No |
| symptom_polyuria | Predictor | 168 | Rejected | No |
| symptom_conjuntival_suffusion | Predictor | 283 | Rejected | No |
| vital_Pressure_systolic_admission | Predictor | 258 | Tentative | No* |
| vital_Pressure_diastolic_admission | Predictor | 254 | Tentative | No* |
| vital_HR_admission | Predictor | 243 | Rejected | No |
| vital_RR_admission | Predictor | 219 | Rejected | No |
| MAP | Predictor | 248 | Confirmed | Yes |
| vasopressor | Predictor | 236 | Rejected | No |
| diuretic | Predictor | 234 | Rejected | No |
| blood_transfussion | Predictor | 232 | Rejected | No |
| antibiotics | Predictor | 295 | Rejected | No |
| lab_urea | Predictor | 259 | Tentative | Yes |
| lab_dela_urea | Predictor | 144 | Rejected | No |
| lab_creatinine | Predictor | 277 | Rejected | No |
| lab_sodium | Predictor | 238 | Tentative | Yes |
| lab_potassium | Predictor | 255 | Rejected | No |
| lab_bicarbonate | Predictor | 151 | Rejected | No |
| lab_pH | Predictor | 155 | Rejected | No |
| lab_bilirubin_D | Predictor | 209 | Tentative | Yes |
| lab_bilirubin_I | Predictor | 208 | Rejected | No |
| lab_TGO | Predictor | 242 | Tentative | Yes |
| lab_TGP | Predictor | 240 | Rejected | No |
| lab_HTc | Predictor | 283 | Confirmed | Yes |
| lab_Hb | Predictor | 272 | Confirmed | No+ |
| lab_leukocytes | Predictor | 284 | Tentative | Yes |
| lab_platelets | Predictor | 284 | Confirmed | Yes |
| length_stay | Predictor | 262 | Rejected | No |

Total predictors: 63; Total predictors Retain: 11

We retain to model training the Confirmed and Tentative predictors.

* systolic and diastolic pressure at admission because we derived the mean arterial pressure (MAP)

+We removed collinear predictors (lab_Hb)

**S 4 - Figure 1.** Confusion Matrix of the results of the xgBoost (A) and the Lasso model (B) in the validation cohort.


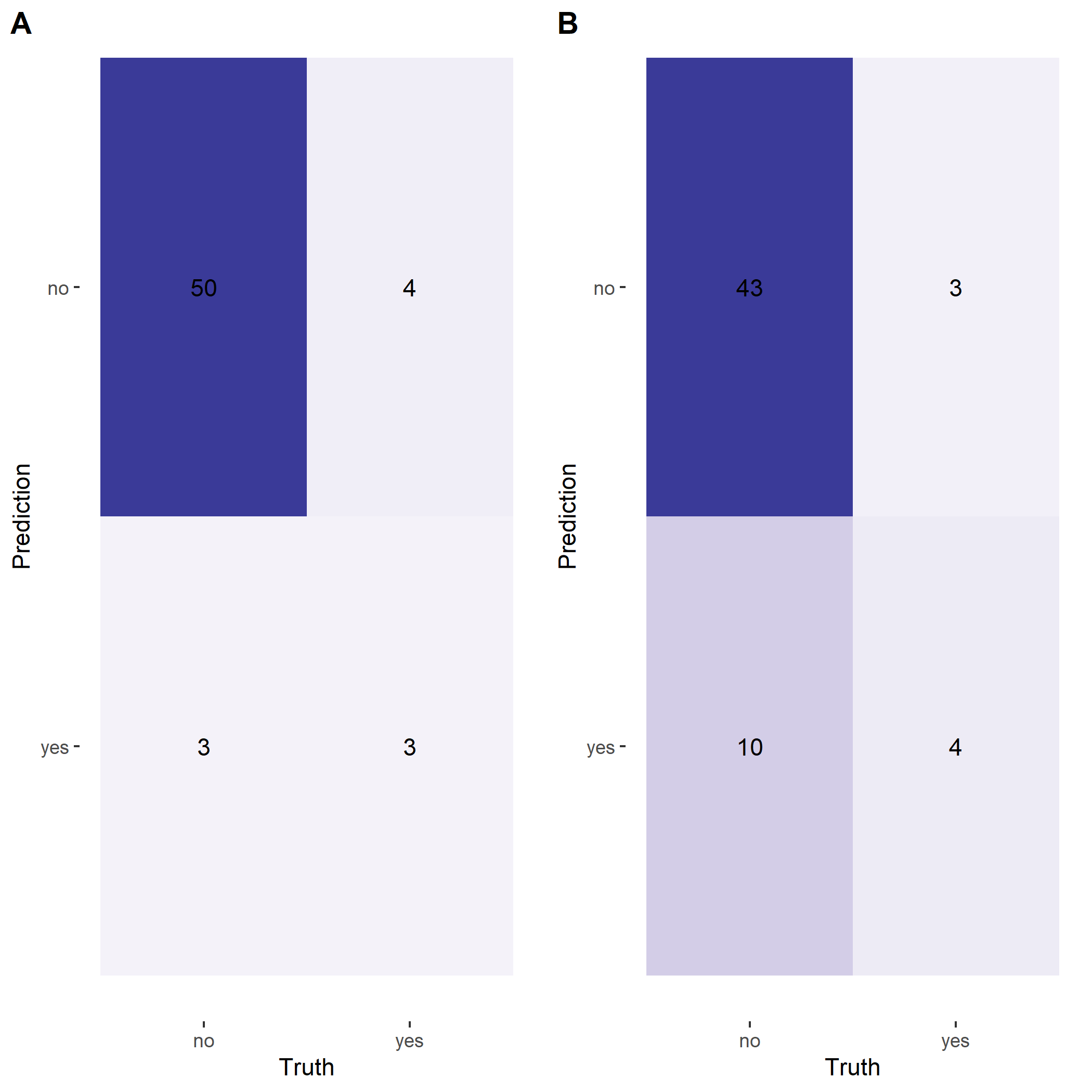


**S 5 - Figure 2.** Variable importance associated with death in leptospirosis patients (Lepto Score).


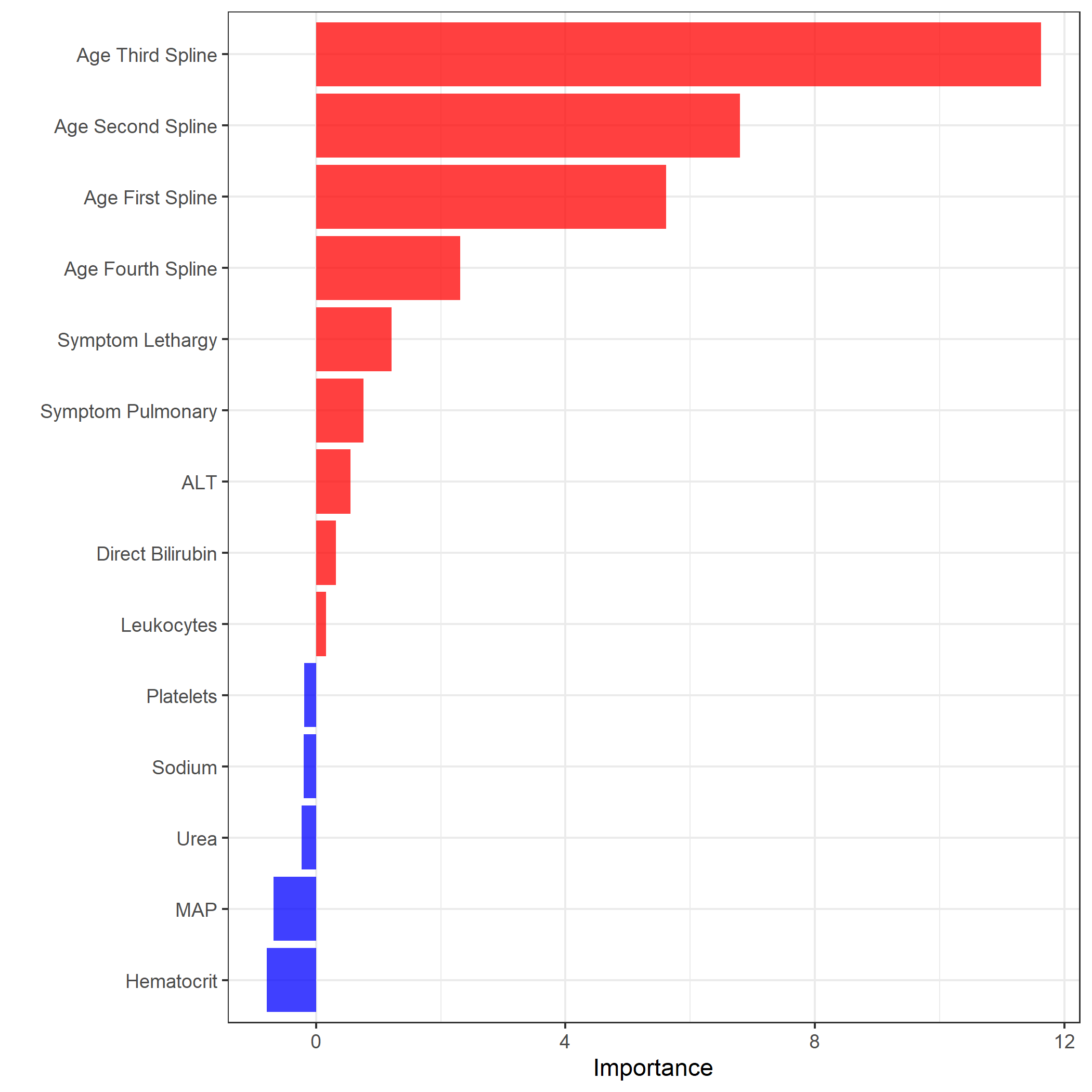


**S 6 - Figure 3.** Classification And Regression Trees for Machine Learning (CART) Plot


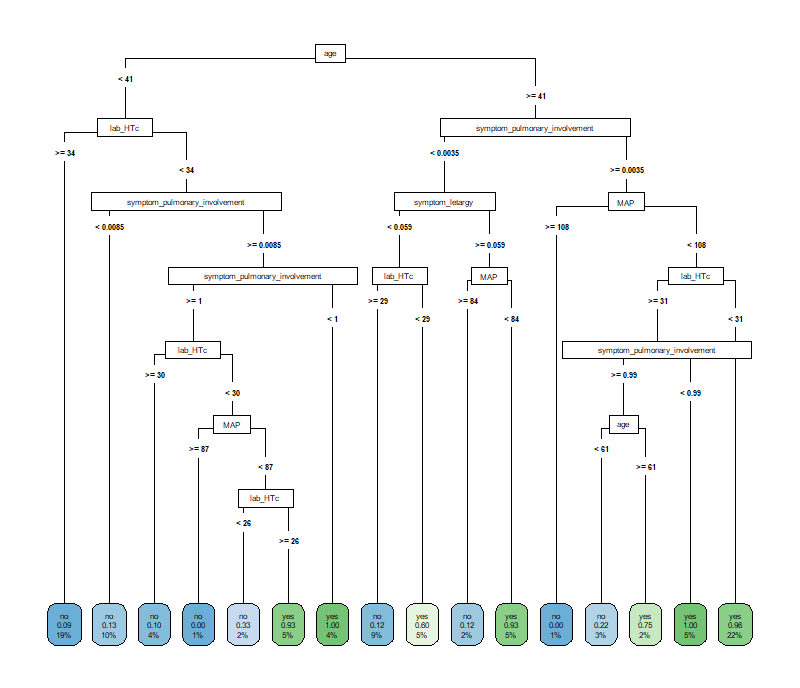


**S 7- Table 4.** Coefficients of Lasso regression (final model)

| term | estimate | penalty |
| --- | --- | --- |
| (Intercept) | -1.6626800 | 0.0158489 |
| Letargy | 0.5782276 | 0.0158489 |
| Pulmonary involvement | 0.6241528 | 0.0158489 |
| MAP | -0.6098900 | 0.0158489 |
| Lab urea | 0.0000000 | 0.0158489 |
| Lab sodium | 0.0000000 | 0.0158489 |
| Lab bilirubin D | 0.3628840 | 0.0158489 |
| Lab TGO | 0.4899103 | 0.0158489 |
| Lab HTc | -0.6401537 | 0.0158489 |
| Lab leukocytes | 0.0000000 | 0.0158489 |
| Lab platelets | 0.0000000 | 0.0158489 |
| Age ns_1 | 0.0000000 | 0.0158489 |
| Age ns_2 | 3.4625012 | 0.0158489 |
| Age ns_3 | 0.0000000 | 0.0158489 |
| Age ns_4 | 0.5815910 | 0.0158489 |

Best penalty was selected by bootstrap in train set.

Estimate was the coefficients of Lasso regression

For Age we use natural splines (ns) with 4 degrees of freedom

To fit the QuickLepto we used the predictors with higher estimate values (in modulo)
